# Body-Mapping the Affective Politics of Bariatric Surgery

**DOI:** 10.1177/19408447251334614

**Published:** 2025-04-21

**Authors:** Meredith Bessey, Carla Rice

**Affiliations:** 1 3653 Department of Family Relations and Applied Nutrition and the Re∙Vision Centre for Art and Social Justice, University of Guelph, Canada

**Keywords:** bariatric surgery, fat studies, arts-based research, body-mapping, affect, new materialism

## Abstract

Bariatric surgery (or weight loss surgery, WLS), an increasingly common intervention into “obesity,” remains a contentious topic amongst obesity experts, critics, and fat activists. As part of a larger study employing a neomaterialist framework, we worked with four women who resided in Canada and had WLS a minimum of one year prior to create life-size body-maps representing their pre- and post-surgical experiences. As a method, body-mapping can bring attention to somatic, embodied, and affective elements, uncovering structures of feeling informing/shaping WLS experiences. We used an affective analytic approach to make sense of the body-maps, which we present according to three affective strands: shades of gray, sensorial-cognitive relationalities with food and body, and entanglements of anticipated and unruly sensations and affects. Body-maps highlight the affective politics that are set into motion by, and set into motion, WLS and the hegemonic discourses and unruly affects that emerged.

## Introduction

As body size becomes increasingly medicalized via “obesity” discourses, bariatric surgery (or colloquially, weight loss surgery; WLS) has become more widely advocated for and accessible in Canada (Obesity Canada, 2019). Yet WLS is a subject of much contention: on one hand, obesity experts advocate for surgery as a “gold standard” treatment, and on the other, fiscally conservative and fatphobic opponents of universal access to WLS argue that candidates are “lazy” and “taking the easy way out” (MacLellan & Johnson, 2021; Trainer et al., 2017). Importantly, fat activists and critical researchers further critique WLS procedures for harms caused to fat people and for buttressing fatphobic rhetoric and praxis in healthcare (Brown & Herndon, 2020; Friedman et al., 2019). Concerns about harms are well-founded, given a litany of studies on the prevalence of negative surgical outcomes including substance abuse, self-harm, and malnutrition (e.g., Castaneda et al., 2018; Thereaux et al., 2019). However, negative experiences are not universal, with many WLS recipients expressing more complex and ambivalent emotions, including satisfaction (e.g., Liu & Irwin, 2017). In our research, we took interest in exploring multifaceted emotive, sensorial, and visceral experiences of WLS using body-mapping, an arts-based approach which can elicit affective responses for both maker and viewer, making it highly suited for exploring embodied topics such as the affective politics surrounding WLS (e.g., Cook, 2022; Fullagar, 2021).

Methodologically, body-mapping originated to examine experiences of people with HIV (de Jager et al., 2016; Solomon, 2007) through a “process of creating body-maps… to visually represent aspects of people’s lives, their bodies and the world they live in” (Gastaldo et al., 2012, p. 5). Researchers have since used it to study various health and body-related topics like sexual health, pain, and health literacy (e.g., Cook, 2022; de Jager et al., 2016; Gastaldo et al., 2018). Typically, investigators pair body-maps with other data generation tools, such as focus groups, turning art-making into a research methodology (Gastaldo et al., 2018); and practitioners also use the approach for advocacy and therapy (e.g., de Jager et al., 2016). Importantly for our purposes, body-mapping offers a way to capture embodied experiences and storied reflections that may not be elicited in interviews or focus groups (Cook, 2022; de Jager et al., 2016). In bringing attention to the somatic and affective, body-mapping allows for a presencing and tracing of the centrality of affect to collective knowing and hence, meaning- and sense-making of WLS.

Though a relatively large body of qualitative research about WLS exists (albeit limited in its theoretical scope and ontological approach; e.g., Aarts et al., 2017; Jumbe & Meyrick, 2018), investigators have generated little arts-based research on the topic. To our knowledge, researchers have conducted only one body-mapping study in Canada that explored perceptions of WLS amongst adolescents labeled as “severely obese” who were contemplating bariatric surgery (Farnesi, 2020); and we have not encountered any arts-based studies about WLS in adults. The use of body-mapping in fat studies generally is also limited. Cook’s (2022) body-mapping study exploring fat people’s intergenerational, affective body narratives included a participant who had undergone bariatric surgery, which they experienced as traumatic and represented visually by drawing silver scars on their body-map and incorporating gold lightning bolt shapes to represent the emotional and physical pain surgery caused.

We took up body-mapping as one component of a larger study consisting of three methods: (a) Interviews with WLS recipients who had had surgery one or more years ago; (b) A body-mapping workshop with a sub-set of interview participants; and (c) Key informant interviews with dietitians who work or had worked in bariatric surgery. The larger study focused on exploring experiential understandings of WLS and employed a feminist neomaterialist framework that promotes a monist ontology, challenging traditional distinctions between scientific and critical perspectives to illustrate how discursive and material forces interact in intricate ways to shape WLS experiences (Rice, 2014; 2015; Rice et al., 2020). Neomaterialist scholars view fatness as something that emerges through ongoing, dynamic interactions with a vibrant sociomaterial environment, rather than as a condition to be pathologized, a perspective that acknowledges matter’s (including fat’s) agency in making reality (Colls, 2007).

Feminist neomaterialism also emphasizes how the research approaches we take enact particular “cuts” that shape and inform what emerges from a project (Barad, 2007); body-mapping is one such “feminist new materialist intra-vention” (Fullagar, 2021, p. 57) that can provide a way for participants to visualize and to bring into consciousness—to register as happening—embodied affects and associated discourses, and the relationalities between them (e.g., de Jager et al., 2016; Rice et al., 2022). We orient to body-mapping as a research method whose agentic “cuts,” in foregrounding/focusing on sensation and embodiment, allow us to register and center affect in our analysis, whereas our interviews emphasized discourse by nature of the different modes of expression. Bringing the “lived feeling body” into view enables us to examine “how subjective experience and biological processes interplay with the social values, political relations, and body concepts embedded within contemporary food regimes and their attendant public health initiatives” (Abbots et al., 2020, p. 358), including within the WLS complex.

## Theoretical Framework

Our theoretical approach considers the politics of affect, which we understand as encompassing the entangled somatosensory, emotive, and sociomaterial dimensions of felt experience. Following Labanyi (2010), we orient to affect as “preconscious and prelinguistic” bodily flows and sensations that exist prior to us being able to name them as emotion (p. 225). To highlight affect’s material dimensions, we pull from Hayes-Conroy and Hayes-Conroy (2008) whose use of the “visceral” to refer to “internally felt sensations, moods, and states of being” (p. 462) allows us to capture sensation’s interactivity with emotion and cognition. We viewed body-mapping as a pathway to explore affect’s political work in the context of bariatric surgery, including via before/after narratives of bodily self-transformation that get constructed in relation to fatness and WLS. Our analysis surfaces “structures of feelings,” a term coined by cultural theorist Raymond Williams (1977), that we argue accompany dominant WLS discourses and regulate people’s decision-making about it. Analytically, we also attend to contrary affects emerging in participants’ accounts that trouble the feeling structures co-constituting official WLS narratives, which we suggest create openings for meaning-making otherwise—for analyzing how in the context of a body-mapping group, for example, participants uncovered and questioned the affects conventionally narrated as attached to post-WLS experiences (e.g., happiness and freedom). We consider body-mapping as an affective practice where space is provided for affectivity, and specifically unruly affects, to be “collaboratively produced” (Wiesse, 2019, p. 131) and articulated, providing participants with more agency over those affects that do not neatly align with dominant structures of feelings.

The concept of structures of feeling has been used in a variety of inconsistent ways since its introduction; here we use it to “name the simultaneously cultural and discursive dimension of… experience” while not neglecting how “experiences are also felt and embodied,” thus allowing “a bridge between language and embodiment” (Zembylas, 2002, p. 194). We take up structures of feeling in our analytic work to surface the taken-for-granted patterns of emotions and sensations that get produced in the confluence/confrontation between differences marked as pathological (fatness) and a normative social order into which difference is symbolically-materially made to fit (via fat shaming, dieting, weight loss procedures, etc.; Boler, 1999). In an anti-fat context, dominant discourses and material arrangements activate certain structures of feeling; in other words, discourse and associated material arrangements structure and mobilize affect for particular purposes. The sociomaterial experience of not fitting in the world as a fat person creates and amplifies affects of shame, embarrassment, self-loathing, etc. (e.g., Meleo-Erwin, 2015). We understand these feeling structures as political insofar as they tether anti-fat affects to epistemic claims in order to influence people’s embodied experiences and relationalities; and, viewed through Ahmed’s (2004) affective economies, these patterns of thinking-feeling, or feeling structures, circulate, flow across, and stick to bodies, making affect into an embodied force that power mobilizes (via markets, medical regimes, public health, etc.) to sway and urge certain action (Rinaldi et al., 2020). In the WLS context, feeling structures attach affects of shame, self-hate, and despair to fatness, and joy, desire, and satisfaction to the possibility of excising or reducing it (e.g., Friedman et al., 2020; Rinaldi et al., 2020). Surfacing these affective structures enables us to understand how they buttress anti-fat discourse/materiality to shape people’s WLS decisions, experiences, and interpretations.

We supplement our analysis of structures of feeling with affective practice to foreground individual’s affective agency, wherein affect is a “dynamic process,” emerging from the intra-actions of bodies, social interactions, discourses, and material arrangements (Wetherell, 2015, p. 139). This consideration of affect enabled us to register other, more unexpected, even recalcitrant feelings and sensations—like outrage, anger, despair, and traumatic wounding that fatmisia and sometimes the surgery itself produced—which despite showing up in participants’ maps, often remain sidelined or ignored in hegemonic “*feels-as-if* stories,” the official narratives about expected before/after affects accompanying surgical procedures like WLS (Hochschild, 2016, p. 135). There is no pre-existing structure of feeling for such sidelined affects; this research is beginning to create it. Following Zembylas (2023), we wanted to trace what these unexpected affective experiences mean and do socially, materially, culturally, and politically. What might, for example, be the impact of giving space for difference-affirming feeling structures to emerge, by bringing people together and providing space for them to engage in affective practice, to discover for themselves what affects they have attached to their WLS experience (Wetherell, 2015)? With this, we ask: what is enabled and foreclosed via the affects and visceral sensations stirred up by both WLS and by the body-mapping process itself? Bringing the visceral and the affective into the analysis allowed us “to account for the complex interplays between lived corporeal materialities and political… dynamics” that are ever-present in conversations about WLS (Abbots et al., 2020, p. 348).

## Methodology and Methods

Ethics approval was received from the University of Guelph Research Ethics Board (file #22-09-018). Participants were recruited for the broader study online, through social media and our existing professional networks. Meredith conducted interviews with participants between March and June 2023; 17 of 21 participants expressed initial interest in participating in body-mapping. We carefully considered who to invite for the workshop, given the group nature of the method and the sensitivity of the topic, and extended invitations to nine participants. Of those, two people declined, one did not respond, and six confirmed interest. Of those six, one had to withdraw due to scheduling issues and a second participant withdrew during the first week of the workshop due to illness, leaving four participants. All participants were women who had had surgery in Canada between one and eight years prior to the study, and who had had a range of experiences post-surgery in terms of success. Participants lived in three Canadian provinces, necessitating an online workshop; our research center has also continued to host many research activities online, in light of the COVID-19 pandemic. Each participant was sent a package of supplies before the workshop, which included two large pieces of white craft paper (that participants could tape together if one piece was not large enough, to attenuate potential access issues), acrylic paint markers, feathers, glitter and glitter glue, pencil and wax crayons, construction paper, tape, and glue. Participants also received $25 to purchase additional supplies and were provided a $50 honourarium for each workshop session they attended, up to a total of $200.

Body-mapping occurred across four weeks in fall 2023, with group meetings taking place on Zoom; sessions were co-facilitated by Meredith and a colleague with experience facilitating body-mapping. Meredith developed an initial body-map facilitation guide based on previous research from Cook (2022); Solomon (2007), and Underhill (personal communication, June 28, 2022) and in dialogue with Carla, which we updated according to initial interview findings, conversations with peers, and the literature. Prompts centered affect and embodiment wherever possible, as highlighted in Table 1.

**Table 1.** Overview of Body-Mapping Sessions.

Session	Focus	Example Prompt/Activity	Length of Session
1	Introductions; establishing ground rules^a^; discussing research purpose/process; and providing instructions on having someone (e.g., family member) trace body-map outline for next session	5-4-3-2-1 grounding exercise (e.g., 5 things I can see, 4 things I can feel, 3 things I can hear, 2 things I can smell, and 1 thing I can taste)	90 minutes
2	Journaling in response to general prompts; discussion about colors and shapes; and start creating body-map in response to prompts 1 and 2	Have the messages that you have received about your body changed over time and/or since undergoing WLS? How could you represent those changing, fluctuating messages on your map? What feelings or emotions come up for you in relation to those changing messages? Where/how might you represent those feelings on your map?	150 minutes
3	Continuing to create body-maps in response to remainder of prompts	Think about the healthcare providers you have encountered (both in relation to surgery and outside of surgery)—how might you represent these people in your body-map? What colors, symbols, or images might you use? What did your encounters with these providers look like, sound like? How did those encounters make you feel? Where do those feelings live?	150 minutes
4	Sharing circle/debrief to discuss content and process-oriented experiences; viewing body-maps as a group; explaining next steps; and workshop closing	How are feelings expressed in your body-map? Where do you imagine these feelings sitting in your body? Do “positive” or “negative” feelings both appear on your body-map? Where do they each sit in your body?	120 minutes

^a^Ground rules included avoiding specific discussion of weight loss numbers, and re-orienting to “fat” as a neutral descriptor of bodies (while acknowledging and discussing how neutrality around fatness is difficult in a fatphobic society), as well as keeping what was shared in the space confidential.

Sessions were recorded and transcribed using automated Zoom transcription, and Meredith then edited transcripts for accuracy and clarity. Meredith and her workshop co-facilitator also held a debrief meeting at the end of each session, and wrote a memo about key events or important/impactful moments from each workshop at the end of each. Each participant photographed their body-maps and emailed the images to Meredith after the final workshop. Using direct quotes from the workshop transcripts, she then created a summary of each body-map, which was shared with the participant who could add, remove, or correct information. Any revisions or additions made by participants were minor. All body-map images, memos, meeting transcripts, and body-map summaries were then uploaded into NVivo for analysis.

### Reflexivity

From a new materialist perspective, this study is itself an intervention into reality and makes waves in the world. The workshop created a space for people to (a) uncover the emotions and visceral sensations that exist, sometimes in the absence of language, around surgery; and (b) understand WLS and their experiences outside of flat questions, such as “is the surgery good or bad?” or “are people satisfied?” By doing this research, we intervene in the field; trustworthiness or value of the research is thus not about replicability, but about noticing the ways we are intervening and thus how our research apparatus might bring something new to the fore (Rice et al., 2022). For example, we consider our creation of a relaxed space, where participants could turn their cameras on and off and engage how they were best able, an agentic cut enacted by this research (Jones et al., 2025; LaMarre et al., 2021); it was important to give participants control over their space so they did not feel surveilled or on display, and to bring people more into the embodied experience than the visual, perhaps leading to different work. Our interest was also in shifting the reality that flattens discourses related to WLS; in thickening our understanding of these procedures, we wanted to better support people’s decision-making and their post-surgical journeys, which necessarily included questioning the goodness or rightness of WLS and suspending moral certainties around these procedures.

One element of the research apparatus that serves to intervene in the given reality is our position as researchers. We come to this topic as two academics who straddle the worlds of theory and practice: Meredith is a 34-year-old, white, queer, thin, cis woman, who is trained as a dietitian and has experienced eating and body distress which she is mostly recovered from, and Carla is a 62-year-old queer fat/not-fat/fat-again femme scholar who once worked as an embodiment therapist having had her own encounters with embodiment/eating distress. This article is part of research Meredith conducted for her doctoral dissertation with Carla as her supervisor. Given our shared experiences at the nexus of theory and practice, we take a fat studies approach that recognizes critical issues surrounding the uncertain/adverse effects of and pressures to undergo WLS whilst holding space for diversity and complexity of lived experiences. Though neither of us have the specific experience of WLS, our lived experiences give us uniquely embodied understandings of bodily volatilities, fluxes, and flows surrounding bodily difference and intentional body modification. Our heightened sensitivities to the affects and visceral sensations that embodying non-dominant and unwanted difference invoke allows us to engage with emotions and embodied elements others without such experiences might overlook; it also allows us to work with the affective and visceral dimensions of participants’ experiences in a workshop that invites bodily vulnerability.

### Analysis

Our approach to analysis was exploratory, as analytic approaches for body-maps are still under development (Orchard, 2017); analysis was informed by Rose’s (2023) approach to compositional interpretation, Guillemin’s (2004) work on analyzing drawings, and Jackson and Mazzei’s (2012) thinking with theory. We oriented to analysis as affective, tracing the flow of affects conveyed through the layering of color, texture, shape, movement, text, and image within the body-maps (Ahmed, 2004; Rice et al., 2021). Meredith began the analysis by reviewing all study data in NVivo, making notes of elements that stood out and beginning to code relevant text segments or visual/textual elements of the body-maps. She developed initial themes (or strands) out of this analysis, which was developed through discussion with Carla and engagement with relevant literature. The themes were developed further as we began writing and developed a greater understanding of the maps and their central elements, and we identified illustrative body-maps for each strand. The body-maps are not simply images but represent an entanglement of the four participants’ embodied and emplaced experiences and contain words, textures, and colors that evoke moods and feelings for readers/viewers; we present the body-maps first to allow them to speak for themselves before applying our own interpretive lens.

## Findings and Discussion

We present findings organized into three affective strands: shades of gray, sensorial-cognitive relationalities with food and body, and entanglements of anticipated and unruly sensations and affects. For each strand, we present one or two body-maps, introduced with a brief description of the participant and their WLS experience and followed by our analysis of the map. The body-maps we have chosen to illustrate each theme provide a vivid illustration of particular affective experiences and politics of WLS, though we see evidence of each theme operating in all body-maps. The analysis is further contextualized with quotes from participants’ interviews, the body-mapping workshop, and/or their body-map summaries. We frame our analysis around the entanglement of emotions, affects, and embodied/visceral sensations foregrounding how body-maps set into motion both dominant structures of feeling as well as resistant/unruly affects.

### Shades of Gray

Gray areas and the color gray were prominent in the body-maps, reflecting the entangled, liminal, and nuanced knot that many WLS recipients find themselves in. We present Deborah and Liz’s maps as vibrant illustrations of this theme.

#### Deborah

**Figure 1.**
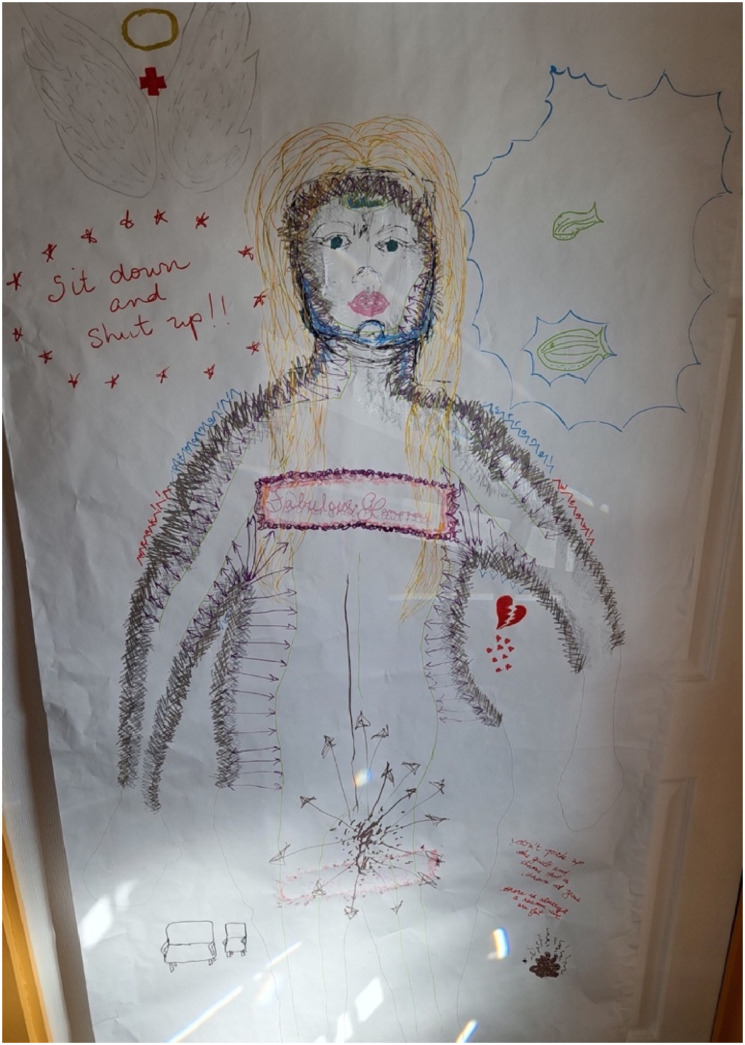
Deborah’s body-map. Deborah's body-map features a feminine-presenting person with long yellow-blond hair outlined with thick grey-black strokes representing her skin. From the thick strokes, double-sided arrows point inward towards the inside of her body and outwards to the world. A small red broken heart floats just outside and to the bottom right of the figure and the words “sit down and shut up” appear on the upper left-hand corner of the image in large print. Large angels' wings and a red cross appear above the words. The bottom left-hand corner of the image features two chairs, one small and the other large-sized. Featured inside the figure, perhaps in her stomach area, are many black dots with arrows pointing outwards.

Now in her late 50s, Deborah had Roux-en-Y surgery (a procedure in which the intestines are re-routed to bypass a portion of the stomach; Piche et al., 2015) approximately eight years before the workshop. While the initial surgery went well and she reached her goal weight successfully and felt “strong and fit and fabulous,” she eventually began dealing with post-surgery complications, including a near-fatal bowel adhesion and severe hernias, both of which caused her to become, in her words, suicidal. (Figure 1)

##### Deborah: Analysis of Body-Map

One of the most striking elements of Deborah’s body-map is the border around her body, which she described as a “frisson”:

You can see on the outside is a frisson of gray and on the inside, there's a frisson of silver because a big reason why I got the surgery is because my outside did not match my inside, and I also wanted to be able to live to 100 and run after grandchildren.

Working with the idea of frisson, defined as “a sudden feeling of excitement or fear” or sometimes a thrill (Cambridge University Press, n.d.), Deborah suggests that their bodily boundaries have become a site of intense sensation, energy, or socio-physio-psychic contestation. Their use of “frisson” points to the entanglement of affect, embodied and physiological sensation, and politics; the boundary between body and world is an active space within which people do intense affective work trying to fit their embodied experience and being, physically, psychically, or morphologically into dominant narratives and sociomaterialities (Ahmed, 2004).

Inside her current body’s borders, Deborah also drew a smaller outline in green with double headed arrows connecting the two layers. Arrows emphasize the fluidity and instability of change and the blurry spatial-temporal boundaries between one’s “before” and “after” selves. The intensified affective work (both sensory and emotive) at the body-world boundary demonstrates how people continue to misfit physically/affectively/sensorially following WLS (Garland-Thomson, 2011), where their body still does not fit perfectly in a fatmisic world. We also interpret the arrows as representing pressures exerted on people in larger bodies to abide by certain social standards and literally compress themselves to fit bodily norms; we see affect as something with physical force, as an energy that is always moving and doing (e.g., Ahmed, 2004; Labanyi, 2010). Deborah’s “frisson” is also reminiscent of a barbed wire fence; the world literally traps people within their fat bodies, by interrupting their movements and sense of oneness with it. Being trapped is both a material and affective experience, with discourses placing the blame for that trap on the body itself rather than on the world’s failure to accommodate it, which many large (and small) bodied people internalize (e.g., Trainer et al., 2022). Dominant narratives about WLS ignore or sideline how surgery creates another trap by leaving WLS recipients in an existentially liminal space where they remain outside the bounds of the species typical human (Apostolidou & Sturm, 2016).

Deborah’s use of the color gray in this part of their map is also meaningful, as they described in an early workshop when brainstorming ideas for their map: “…because I went from, you know, absolute… success to now, I'm not considered successful anymore… so, a little bit of gray around the edges, so, but spectacular in the center, so, yeah.” The post-WLS space is a gray area, an area where there is little scientific or medical certainty. Qualitative literature describes uncertainty as a central component of many people’s WLS experiences (e.g., Natvik, 2015); and significantly, how the body responds to this procedure remains a liminal space even in the clinical literature. For example, Cadena-Obando and colleagues (2020) note that predictors of surgical success remain uncertain, and Ward and Ogden (2019) describe bariatric surgeons’ uncertainty about whether complications pre-existed or were caused by the surgery. There is a disconnect between the hegemonic attempt by the WLS apparatus to impose certainty and the reality that surgical outcomes are often anything but certain; this disconnect leaves people in a state of what we might call “affect trouble” where they are left struggling to embody the often-elusive WLS success story. For example, whilst the hospital where she underwent surgery made Deborah into a WLS poster child by featuring her in its PR materials, by the time it published her profile, Deborah was already “feeling a little bit of a fraud” as she had begun dealing with adverse health consequences related to the intervention. In their body-map, Deborah embodies shades of gray despite the WLS system having used their intense bodywork to tell a story that matches the success narrative, a narrative they no longer align with because their body is acting back in ways no mind can control.

#### Liz

**Figure 2.**
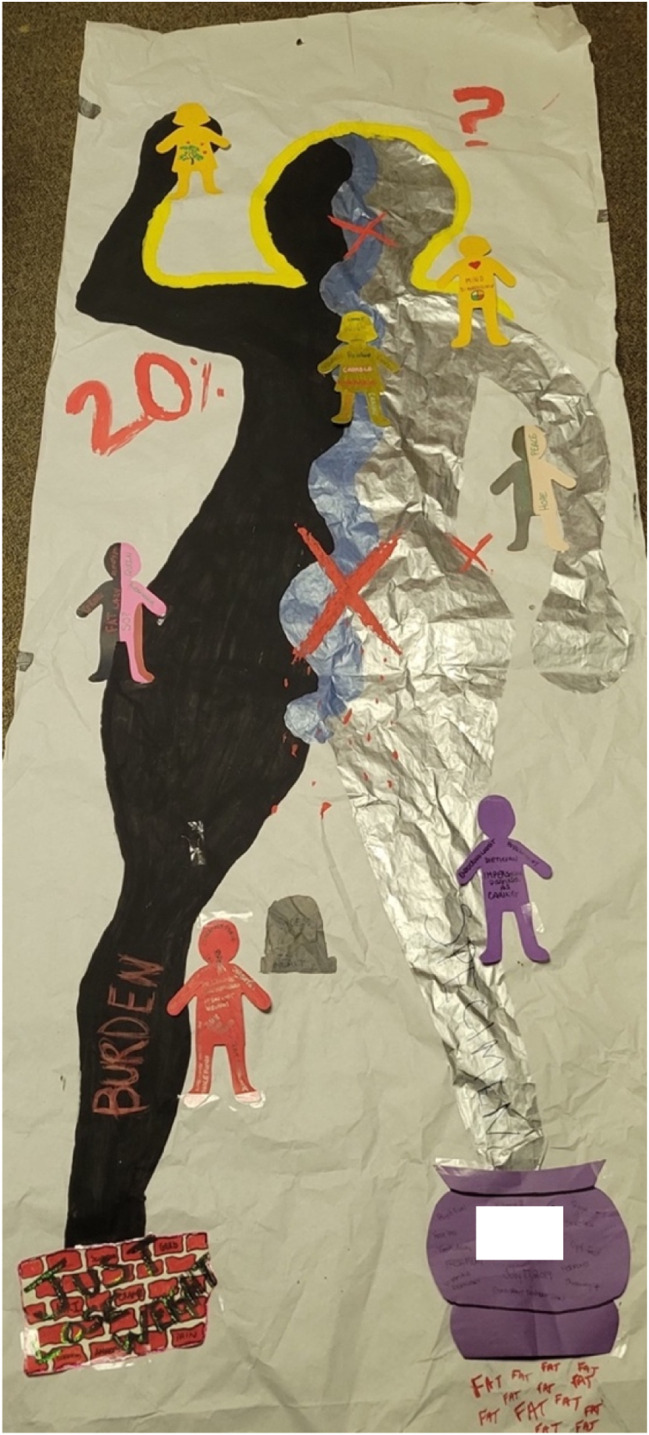
Liz’s body-map. Liz's body-map features the ouline of a person with one arm pointing up and one arm pointing down. The left side of the figure is coloured solid black while the right side is silver, with the two sides connected by a blue wave through the centre of the figure. The left leg says “burden” in large red letters, while the right leg says “specimen” in black letters. The figure's head is surrounded by an outline of yellow, and there is a large red question mark to the right of the head and a 20% written in red under the left arm. On the left foot is a brick wall that states “just lose weight” while the right foot has a large purple meat grinder with words scattered across it and the word “fat” written multiple times underneath.

Liz is in her mid-40s and had a sleeve gastrectomy (a procedure in which the stomach is reduced to a narrow “sleeve” about the size of a banana; Piche et al., 2015) approximately four years before participating in the study. She was generally very satisfied with the results of her surgery and was able to access medical care post-surgery that she could not access prior to surgery, but also had major concerns about the WLS system and the care (or lack thereof) that people are provided within it. (Figure 2)

##### Liz: Analysis of Body-Map

As Deborah did above, Liz also centered shades of gray in her body-map, alongside both fluidity and duality, in the way she chose to represent her pre- and post-surgery self:

I've been chewing over the word fat… and the transformation that word had over the course of my life… where I got to this place of acceptance with it and empowerment with it, and then I had this radical change, so, I've been really thinking about that dichotomy that I represented in my map with the two sides… I chose black as my before because that's kind of where I was coming from, from that pit. I'm still in a gray area though, but I used silver because there's still those glimmers. I drew a river in the middle and I tried to make it marbled to show the flow, because there's gonna be flow between the two sides of me since they're still connected, [the before] side still deserves that love.

Though Liz represented the fluidity of her physical embodiment before and after surgery with this rendering, her work also spoke to the fluidity of self and identity. She had a strong connection to and acceptance of herself as a fat person, and she had to navigate a huge shift in identity post-surgery. The gravestone that she drew on her body-map might also reflect this identity shift: when sharing her body-map, she asked “at what cost did I succeed?” Part of the process of going through surgery involved coming to terms with the loss of parts of herself that she (and the procedure itself) left behind and accepting those aspects of her identity that did not and would not change, even with major weight loss. Despite the operations of a cultural split screen clearly demarcating before from after, the “before” version remains present in the “after,” as Liz represented by connecting her two sides with a river. In her map, Liz has created and troubled her own split screen, or the before/after dominant narrative trajectory of WLS (e.g., Ourahmoune, 2017). In addition to interpreting her map as representing the two sides of herself, we can interpret this as the dominant before/after dualistic narrative being disrupted by her more fluid embodied experience, though there is still a desire to fit her experience within that narrative frame. Her experience also grapples with a kind of frisson or area of charge, as with Deborah, but the charge or affective intensity is located in the river where past and present embodiments come into contact and blur and blend together, rather than in the space where her body and the world meet.

Liz’s choice to have surgery originated from several places, including from a desire for adequate healthcare:

I had always said… if my weight ever impacted my access to health care or anything like that, then I would seriously look at the measures I needed to do to rectify that, so I could get proper care, which is a whole other thing that's come up, what is proper care and who deserves it.

On the “before” side of Liz’s body-map, she refers to herself as a burden and uses “gross,” “wrong,” and “lazy” to describe other people’s perceptions of her before surgery. She also represents herself using little cut-outs of people in a few places on her map:

I used muted colours for both these little people, to further show confusion in my journey; some [things] stayed the same but [there’s] still confusion, it speaks more to that dichotomy and those gray areas that fat people live in. They exist in the margins of the medical community.

Liz speaks to unruly or unexpected affects, like confusion, while underlining the dichotomy that is present, where those unruly affects are sidelined, disavowed, or pushed to the margins. She captures the dehumanizing material and discursive barriers that fat people face within the healthcare system, when they are repeatedly told to “just lose weight” instead of being adequately assessed for the health issues they are facing (Friedman et al., 2020), through her depiction of a brick wall on her right “before” foot. We interpret this wall as a metaphor for epistemic ignorance (Alcoff, 2007), speaking to medicine’s imposition of certainty in an uncertain area of human health (e.g., Swierz et al., 2020), where losing weight is reinscribed as the true path to health, despite contestation, complexity, and uncertainty. The brick wall represents the manufacturing of a specific set of truths in a way that dismisses and undermines embodied experiences that run counter to those truths.

After her surgery, Liz was grateful to receive adequate care in some circumstances, but also felt anger at the lengths she had to go to get it:

Liz: And it would have saved me so much needless suffering, and that just speaks to women's health and suffering though in this space as well, but as a person of size… I got no consideration, but now that I'm slim, I'm kind of throwing it back on them…

Deborah: You had to mutilate your body in order to be taken seriously.

Liz: I had to give up my entire identity of who I was and all the work I did to accept myself in order to be valid in the eyes of the medical community, and I'm still working on that, to where I'm here going… now that in your eyes I deserve care, why am I still not getting it? So, I think that they use people being fat as an excuse to dismiss them and their needs, especially in this territory where we have, like, a lack of resources.

For Liz, the affects tethered to fatness circulating in the healthcare system moved her toward WLS in a bid to prove that she is worthy of respect, dignity, and care, and that she could fit. Fat hatred hurts people, but people also resist and act, taking the avenues available to them to escape difficult and impossible situations; the pathways out of the pain of being fat and a woman within the current cultural moment are limited (Rice, 2007), with WLS being one of the more culturally and medically accessible and authorized ones for some subjects in certain jurisdictions including Canadian provinces like Ontario. The surgery did not wholly deliver on its promises, however; Liz still misfits and gets dismissed. Anti-fat affects continue to circulate even now when she is in a smaller body. Medical and cultural narratives of individual responsibilization set particular structures of feeling surrounding fat embodiment and its regulation by systems and subjects into motion (e.g., Zembylas, 2023). Though other structures of feeling, or ways of feeling-thinking, are simultaneously vying to emerge, dominant discourses push them out, allowing fatphobic narratives to dominate affectively, cognitively, and hence, politically. These non-dominant feelings do not always have associated discourse and narrative; without language to describe these aspects of experience, they are abjected or cast aside. In this article, we are in part attempting to create space for and construct a narrative around these insipient feelings where none already exists.

Liz represented this affective and physical wound on the “after” side of her body-map, where she writes the word “specimen” down her leg:

On my after foot, is my… bariatric meat grinder with fat coming out. For me, it was all of this body acceptance that I had really worked at, worked really hard to do, and where does that go now and how can I still use that?

Liz also wrote the words “fresh meat” on the meat grinder itself, where fat is the output. In our interpretation, this is material, in how WLS physically sheds fat from people’s bodies after WLS, and symbolic, in terms of the psychic parts that people leave behind or disregard through the bariatric surgery process. The very viscerally and affectively charged descriptions of herself as a specimen or as fresh meat call to mind a medical experiment; Deborah’s earlier description of the surgery as “mutilation” does the same.

### Sensorial-Cognitive Relationalities with Food and Body

Across the body-maps, workshop conversations, and participant interviews, sensorial (sensuous, affective, and visceral) and cognitive (minded) relationalities with food and with one’s body were apparent, highlighting the entanglement of feelings, physiological sensations, and embodied experiences. We present Shaun’s body-map as an illustration of this theme.

#### Shaun

**Figure 3.**
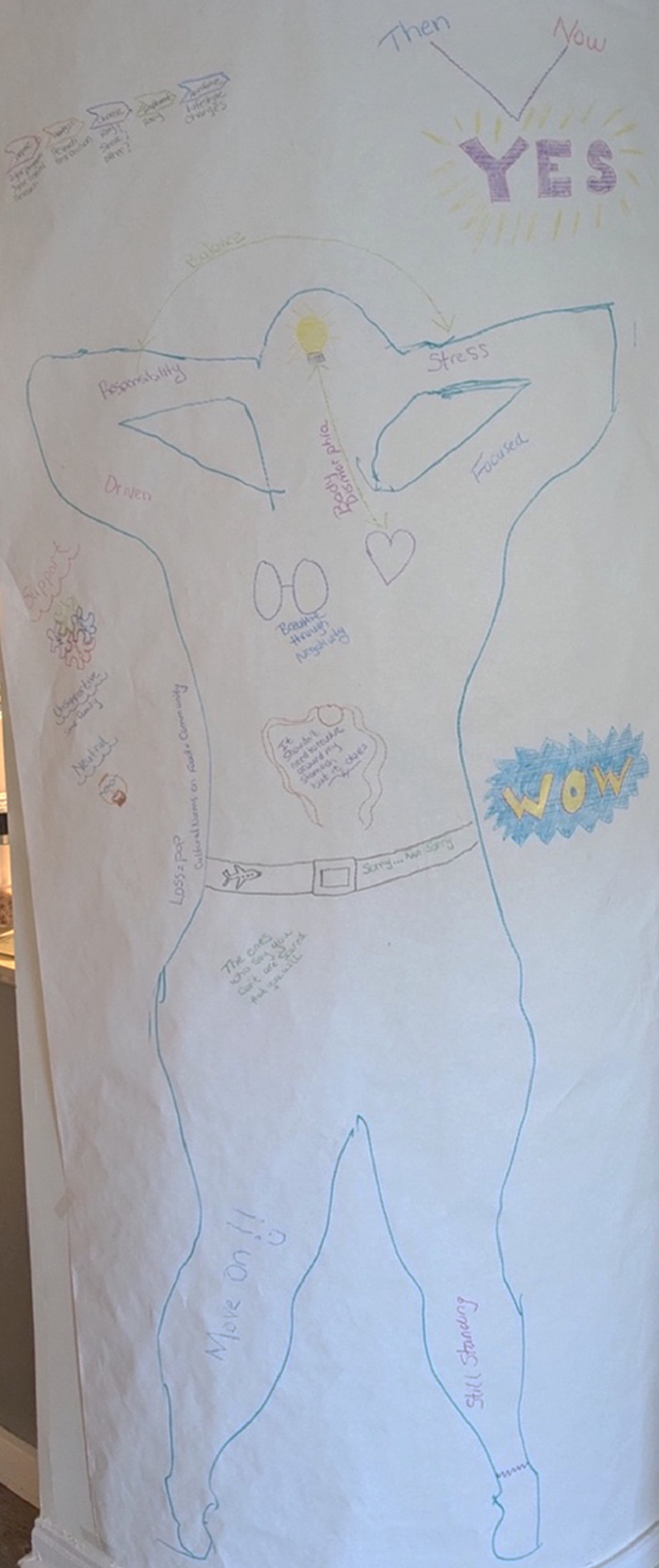
Shaun’s body-map. Shaun's body-map features a person outlined in light blue with their arms lifted so the hands touch the back of their head. On the right side above the figure's head is “yes” written in large purple letters with “then” and “now” written above it, connecting to the word “yes” with a line, and on the left side is a small process chart. Inside the figure's head is a lightbulb connected to an image of a heart by a double-headed arrow next to which “body dysmorphia” is written. On the right side of the figure's waist is the word “wow” in large yellow letters, and around her waist is a belt with the image of a plane and the words “sorry, not sorry”. The right leg says “still standing” while the left leg says “move on!!”

Shaun, now in her early 50s, had Roux-en-Y surgery almost four years prior to the study. She was grateful to the doctor who suggested that she have WLS, and felt very supported prior to, during, and after. At the time of the study, she had recently undergone surgery to remove excess skin. Though she was satisfied with her experience, she expressed concerns more broadly about the bariatric system in Canada especially in provinces where services and supports are limited, such as in Nova Scotia where she (and the first author) lived at the time. (Figure 3)

##### Shaun: Analysis of Body-Map

Embodied affect, sensory relationships with food, gray areas, and other unruly sensations were all evident in Shaun’s body-map. She shared that engaging in body-mapping allowed her to reflect on the root of the changes she experienced and to acknowledge the aspects that were “not a hundred percent positive.” For example, on the left side of her body-map, she indicated losses she experienced:

I was saying to [my husband]… I really miss just being able to overeat at Thanksgiving and I just want to eat a whole box of Stovetop stuffing basically… and I said, like, my stomach just won't allow it and… I miss that and I don't know if I miss the food or if I'm missing… 'cause I still feel comfort, my family’s all there and we're all together, but I just miss unbuttoning my pants cause I'm over-full… so, there is a loss there too, it's just super counterbalanced…

Using food to soothe oneself can become an embodied habitus or a primary way to comfort oneself, especially in a context where eating and food are gendered and a way that women care for themselves and others (e.g., Cairns et al., 2010). There is evidence that people with body mass indices in the “obese” range may have greater sensitivity to food-related sensory stimuli and affective responses (Weltens et al., 2014), meaning that additional support in finding ways to comfort oneself post-WLS is potentially warranted. The affective, sensory importance of food was clear in an exchange amongst Chloe, Liz, and Deborah:

…there's the food that nourishes your body and then there's the food that nourishes your soul… to build a good relationship with those foods is such an important thing. However, that needs to happen for you, you know, so, we're not trading one eating disorder for another. (Chloe)

Liz, in response, noted that ice cream is a soul-nourishing food for her, and explained that

food was so tied into the culture of my family and my friends, we went out to eat, we celebrated with food, we mourned with food… everything was about food. I had to spend five weeks with my mom… and I just saw, like, that food obsession again…

Deborah additionally pointed out the emotional comfort that food can provide:

Liz… you went kind of like this [drawing arms in towards her], like [Liz gesturing], that's it, exactly, that is the feeling, that wrap you in a blanket and give you a hug cause nobody else is [laughing], so.

This conversation highlights how experience, memory, familial experiences, relations of care, etc. all “intersect with individuals’ sensory grasp of the world” to influence their affective and visceral experience of an eating event (Abbots et al., 2020; Hayes-Conroy & Hayes-Conroy, 2008, p. 465). Memories of past events are “materially affective” within a present event, creating sometimes unexpected memory traces and impacts on food choices (Fox & Alldred, 2019, p. 25). People engage in a sensorial, affective relationship with food/eating, and for many people in larger bodies, the world’s perception of their size often disallows them from engaging with food in a pleasurable, sensuous way (Rice, 2007). For participants in this study, achieving a more socially acceptable size has made pleasurable eating experiences somewhat more socially available, but their new digestive anatomy also creates a block. Food plays a physiological role in shaping embodiment, in that the comfort we often seek from it is not just affective and emotional but also encoded in our brains and guts (e.g., Weltens et al., 2014). Despite associated challenges, participants still attempted to soothe their emotions within the boundaries and limitations of their post-surgical body by eating smaller amounts or different types of food or using non-food coping mechanisms. However, some amount of grief and sadness still accompanied this experience for some.

At the center of her body-map, Shaun drew her digestive organs, and reflected on some of her ambivalent feelings related to that depiction:

I'm super hyper-focused on every single thing that goes into my body, and I represented that with what’s supposed to be my intestines with just a little note that it shouldn't need to revolve around my stomach, but it still does three years later, I cannot forget about it, it's a daily task for me that’s just constantly on my mind.

There is a sense that one *should* be more relaxed about food after bariatric surgery, but there is perhaps some guilt or stress when that does not always occur. Shaun cannot divorce her thinking mind from her embodied experiences of eating and remains cognizant of managing a body that has been societally coded as unruly (Chandler & Rice, 2013). She made a similar comment related to weight as a metric of surgical success; while Shaun has a sense that weight *should not* be the focus after going through WLS, it was difficult for her to entirely de-emphasize this outcome. Letting go of weight as a focus is especially challenging when so much of the messaging around surgery is still related to weight loss as a key outcome (e.g., Sanchez-Cordero et al., 2022), despite a supposed desire within obesity medicine to shift toward less weight-centric approaches (e.g., Frühbeck, 2015). From our vantage, this shift might speak to how obesity scientists continue to change the official narrative about WLS as people’s bodies rebel against the surgery’s supposed weight loss outcomes, a line of inquiry we take up in more depth elsewhere (Bessey & Rice, 2025, in preparation).

Shaun articulates the post-surgical embodied shifts that she has undergone:

What I have noticed is my body has shifted, my centre of gravity has shifted, and I feel things in different locations now, and I think that is due to weight loss. So before, responsibility and stress were very much in my gut area and interestingly enough, those have moved more to my upper back and shoulder area as I’ve moved on…

Shaun’s comments demonstrate the embodied nature of affect, and how affect can shift within the body as it undergoes physical changes post-surgery. Affect “lives” in the body, as Liz also illustrated when she commented that “I can feel it in my gut and my chest and my throat, that's where a lot of that's coming out,” in relation to the insights that body-mapping provided; she also noted that “my more traumatic things, I'm carrying in my legs… and associated pain that comes with that,” when thinking about where she carries certain emotions. Body-mapping as an affective practice allows participants to make connections between mind and body, thought and feeling, and to arrive at embodied insights that help to expose affect’s operations in governing subjects within fatmisic, neoliberalized, biomedicalized contexts such as our own. For example, the trauma that Liz references largely relates to the experience of being a fat woman in the world (Rice, 2007), which she described during her interview as “probably one of the biggest traumas of my life”; the affect that body-mapping brought to awareness is produced in context and is entangled with the political. How affect moves *within* the body following a major intervention like WLS is an area for future inquiry.

Shaun’s experience suggests that people might experience significant somatosensory shifts, including in body awareness or proprioception, following major weight loss after bariatric surgery (e.g., Guardia et al., 2013). People’s size awareness also seems to be impacted; for example, Deborah commented that she was very aware of her body size leading up to surgery, but had several colleagues comment after surgery that she would barge into things, demonstrating little awareness of her smaller body in space. Furthermore, many people experience body dysmorphia post-WLS (e.g., Ourahmoune, 2017), which Shaun said she “suffer[s] from greatly”:

…I find it super fascinating from a scientific point of view, how you can know that you're that much smaller, but look in the mirror and really not see it, I cannot see it, and I find that absolutely amazing. A little bit of a wow moment for me was after I got traced, that was the first time that I did not experience body dysmorphia. When I saw my outline, I was like, “Holy crap, I have a waist!”… this picture was the first time in almost four years that I can actually see that, so I think that’s a breakthrough.

The rapid nature of weight loss following WLS may not allow people to update their body schema (Guardia et al., 2013). The experience of body-mapping afforded Shaun with insight and support by allowing her to align her body schema with her current post-surgical body, suggesting that this method may have therapeutic benefits for post-WLS recipients who are feeling disconnected from their body following the intervention.

### Entanglements of Anticipated and Unruly Sensations and Affects

In all four maps, we see evidence of tension between normative and counter-hegemonic stories about fatness and weight loss, as well as between anticipated and non-officially endorsed, unanticipated, and unruly affects. We consider how the body-maps both endorse dominant structures of feeling and set resistant/unruly affects into motion, using Chloe’s map as a vibrant illustration of this thread.

#### Chloe

**Figure 4.**
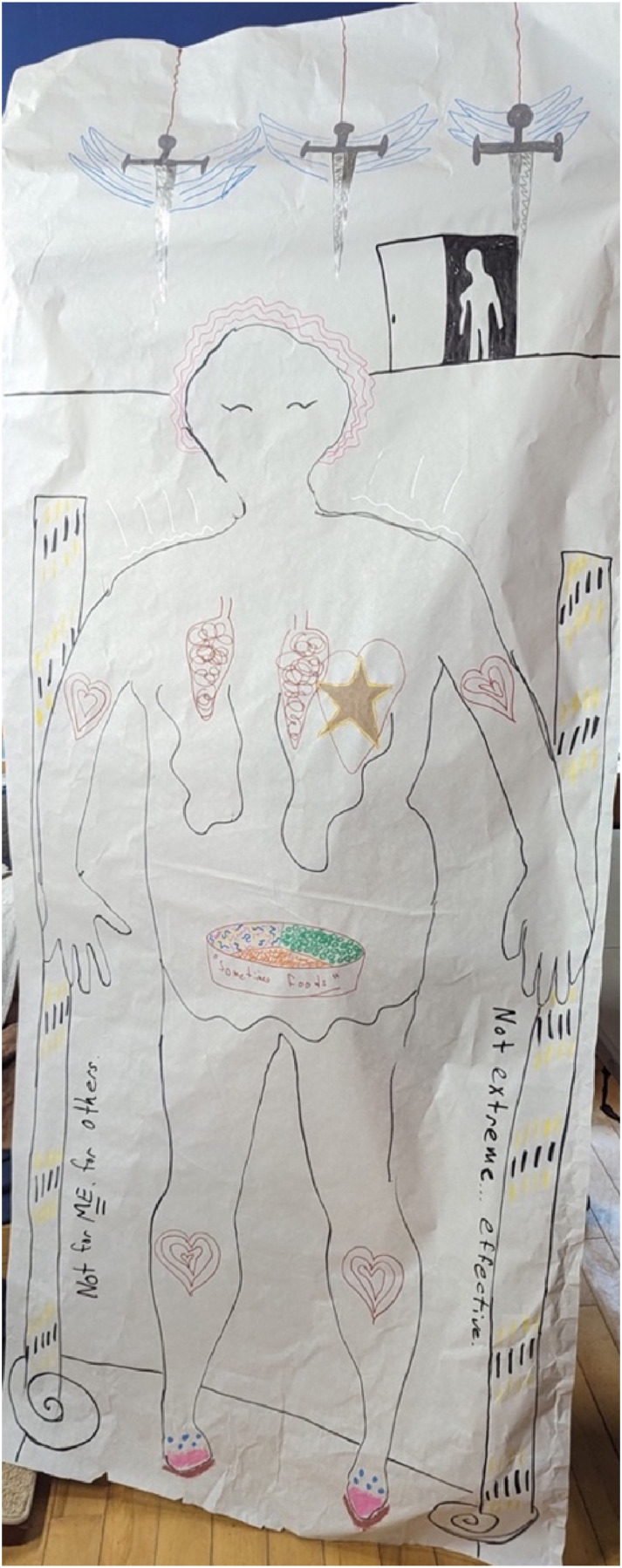
Chloe’s body-map. Chloe's body-map features a feminine-appearing person with pink squiggly lines drawn around their head. Behind and above the person is an open door with a shadowy figure emerging, as well as three swords hanging from red string. In the chest area is a red outline of a heart with a gold star at the centre, and squiggly black shapes. At the centre of the figure, in the stomach area, is a plate divided into three with the words “sometimes foods” on the side. The figure is framed by black and gold bars and the words “not for me, for others” on the left, and the words “not extreme... effective” on the right. There are red, multi-layered hearts drawn on both arms and both legs, and the person is wearing pink shoes.

Chloe is a woman in her early 30s who had a sleeve gastrectomy approximately one year before the study, followed by a revision to a duodenal switch (which combines the sleeve gastrectomy with an intestinal bypass as in Roux-en-Y; Topart & Becouarn, 2017) nine months later. She was generally satisfied with the results of her surgeries and felt healthy and confident in her decision but was also aware that things could still change for the negative in the future. (Figure 4)

##### Chloe: Analysis of Body-Map

Entanglements and tensions between dominant and counter-hegemonic narratives were prevalent in Chloe’s map and narrative. Despite her success following WLS, Chloe challenged dominant before/after narratives, including when she noted that “I’m smaller than I was, but objectively, I’m still obese.” For participants, success was not always defined based on moving into a different body mass index category but rather on how they felt following their surgery and their perception that they were putting in effort to improve their health. Additionally, when a participant commented that they loved the way before and after was (fluidly) represented in Liz’s body-map, Chloe reflected in response:

I agree. It’s interesting, I try not to think about things as before versus after. I’m still the same person. But a before and after mindset is still really helpful and very individual. It’s tough NOT to think of before and after when you live in a universe with a progressive timeline lol.

When Chloe says “I’m still the same person,” she might mean that she has narrative coherence (e.g., Vanden Poel & Hermans, 2019)—expressing an internal consistency across the before/after, where she carries embodied memories of the before though she is living the after. Here, Chloe also comments on the dominant WLS narrative, which reproduces a temporal binary that positions surgery as the critical turning point in people’s life journeys (e.g., Cook, 2022; Ourahmoune, 2017); participants resisted this narrative frame but also used it to make sense of their experiences, as there is no other to rely on. Chloe and others sense and narrate themselves beyond and outside this hegemonic frame because their bodies are insistent and act back; they understand that their stories and experiences do not neatly fit into the expected framework, even if some elements do.

Entanglements and tensions also surfaced in a conversation the group had about food and tracking; Shaun started by noting that she keeps track of everything that she eats, and Chloe agreed that she does the same. In response, Deborah advised that they continue to “do it [and] don't ever, ever, ever, ever stop. The minute you stop [tracking] is when it sneaks back at you, take it from the future.” Liz then countered this advice by saying:

I have to say, I haven't tracked since a year in, and I had to stop because the obsession with the numbers didn't equate to me having balance in my life… so, always make sure if you're counting, you're having that balance part. Just devil's advocate, different experience.

Deborah, Shaun, and Liz then went on to discuss how everyone measures success in different ways, and how it is important for individuals to find balance in ways that feel right for them. In relation to that conversation, Liz shared:

I just think that the conversation we just had was fantastic, it's given me more ideas about that balance piece and the imagery associated with that balance piece, right? Because we do walk that fine line, and I think it was Chloe who brought it up in the beginning, like, there’s two worlds, right? And you're in that gray area and gray’s a huge theme in the colours that I picked too…

Chloe added: “it's finding the balance between being that numbers person and wanting to see everything laid out in a certain way, but also not becoming too unhealthily obsessed about it… yeah, relate, totally.” Maintaining control via tracking is seen as *the* way to be successful (e.g., maintain weight loss) after bariatric surgery, but participants had to find their own way through, both within and outside the dominant story.

Relatedly, Chloe spoke to the dissonant or contradictory space that many WLS recipients come to exist within post-surgery when brainstorming ideas for her body-map early in the workshop:

…I started thinking about the sort of gulf of cognitive dissonance between being fat positive and loving yourself as you are and then also getting [WLS], there's this area in the middle… [that’s] an emotion that defies labeling… not really guilt, not really confusion… but sort of somewhere in that world, it doesn't feel good… but then, it… defies trying to describe that kind of emotion…

Liz, in response, recalled how the medical system wants to make WLS, or weight generally, into a black-and-white experience that can be reduced to a checkbox, even though that is not the reality of people’s lives:

…one of the shapes… I noted down was boxes, how there's so many boxes in this process, um, the colour’s black, um, black and white, checklists, cages, fitting in, different boxes for different sizes, medical mysteries that were not weight related, could have been shown in a checklist that I wasn't afforded when I was a fat person. It wasn't given to me until after surgery, but that checklist wasn't weight dependent. So, I'm thinking of those things and that just kind of came out.

Affects and sensations that exceed the dominant storyline get disavowed, repressed, and othered. Pre-surgery, WLS recipients do not necessarily fit into the boxes that society expects them to, but the bariatric surgery system also exists in a black-and-white space where recipients may again find themselves not fitting into these pre-determined categories or boxes. There is no pre-existing schema where their experiences fit neatly, leaving things muddy or unclear.

Lastly, Chloe’s use of the phrase “not extreme… effective” along the right-hand side of her map is worth explicating. This is seemingly in response to critiques of WLS from both fat activists who view the surgery as harmful and damaging to fat people (e.g., Brown & Herndon, 2020; Friedman et al., 2019), and from people who view the surgery as the “easy way out” and feel that people should diet and use personal control to manage their weight (e.g., Trainer et al., 2017). This statement also connects to what she wrote on the left side of her map: “not for me, for others,” reflecting something she said in her interview: “[WLS] wasn't really on my radar actually, I had never kind of considered it, oh, that's kind of, you know, an extreme thing that people do, but I would never.” Chloe had known a friend of a friend who had died from bariatric surgery and so viewed the surgery as extreme but eventually was motivated to have the surgery by her concerns about her long-term health; her positive experience has seemingly reinforced for her that the surgery is not extreme but is in fact effective. Even so, she, and other participants, were hesitant to recommend this surgery across the board, as she recognized that “it really is not going to make you happy just because you can lose weight” and emphasized that surgery is not “a fix-all magic push of a button,” continuing to resist hegemonic norms about bariatric surgery as a miraculous intervention (e.g., Bombak et al., 2021).

## Conclusion

The affective politics and structures of feeling that set in motion and are set in motion by WLS were evident throughout participant body-maps, including how these affective dimensions entangle with material, visceral facets of WLS experience. Body-maps reflected various discursive, material, affective, sensory, and social aspects of participants’ WLS experiences, specifically in relation to the three affective strands: shades of gray, sensorial-cognitive relationalities with food and body, and entanglements of anticipated and unruly sensations and affects. Body-mapping served as an affective practice wherein WLS recipients could explore embodied aspects of their identities and how undergoing this surgery impacted them—negatively, positively, and in-between. A lack of non-dominant discourse about WLS creates little to no space for people to make sense of their felt experiences; our work begins to create the space for these unruly affects to emerge. This method foregrounds the affective dimensions of WLS experiences in giving participants opportunity to create a representation outside of themselves, which can allow for the critical distance required to self-interpret and may afford therapeutic benefits even in a research context. For example, body-mapping helps to articulate and give expression to new structures of feeling that could be used in therapeutic contexts to help therapists and clients to sense make about clients’ abjected or sidelined experiences. This is an area ripe for exploration in future studies adopting this methodology.
